# The CNVrd2 package: measurement of copy number at complex loci using high-throughput sequencing data

**DOI:** 10.3389/fgene.2014.00248

**Published:** 2014-08-01

**Authors:** Hoang T. Nguyen, Tony R. Merriman, Michael A. Black

**Affiliations:** ^1^Department of Biochemistry, University of OtagoDunedin, New Zealand; ^2^Department of Mathematics and Statistics, University of OtagoDunedin, New Zealand; ^3^Department of Biochemistry, Virtual Institute of Statistical Genetics, University of OtagoDunedin, New Zealand

**Keywords:** copy number variation, read depth, 1000 Genomes, CCL3L1, DEFB4

## Abstract

Recent advances in high-throughout sequencing technologies have made it possible to accurately assign copy number (CN) at *CN* variable loci. However, current analytic methods often perform poorly in regions in which complex *CN* variation is observed. Here we report the development of a read depth-based approach, CNVrd2, for investigation of *CN* variation using high-throughput sequencing data. This methodology was developed using data from the 1000 Genomes Project from the *CCL3L1* locus, and tested using data from the *DEFB103A* locus. In both cases, samples were selected for which paralog ratio test data were also available for comparison. The CNVrd2 method first uses observed read-count ratios to refine segmentation results in one population. Then a linear regression model is applied to adjust the results across multiple populations, in combination with a Bayesian normal mixture model to cluster segmentation scores into groups for individual *CN* counts. The performance of CNVrd2 was compared to that of two other read depth-based methods (CNVnator, cn.mops) at the *CCL3L1* and *DEFB103A* loci. The highest concordance with the paralog ratio test method was observed for CNVrd2 (77.8/90.4% for CNVrd2, 36.7/4.8% for cn.mops and 7.2/1% for CNVnator at *CCL3L1* and *DEF103A*). CNVrd2 is available as an R package as part of the Bioconductor project: http://www.bioconductor.org/packages/release/bioc/html/CNVrd2.html.

## Introduction

Copy number variation (CNV) encompassing genes is a common phenomenon in the human genome, and has been shown to be associated with variation in phenotype (Gonzalez et al., 2005; Freeman et al., 2006; McKinney et al., 2008, 2010, 2012; Bentley et al., 2010). Accurate CN assignment is required for studies associating CNV loci with phenotype. However, accurately measuring gene CN by direct molecular methods over large numbers of samples is challenging, and is often complicated by the existence of paralogous gene pairs. As a consequence, phenotypic association data, largely obtained by quantitative polymerase chain reaction (Q-PCR) based methods, should be regarded with caution (He et al., 2009; Marques et al., 2010; Carpenter et al., 2011; McKinney and Merriman, 2012). In response to the increasing utility of high-throughput sequencing (HTS) technologies, we previously developed the read depth based method CNVrd (Nguyen et al., 2013). We demonstrated its ability to accurately assign CN using genome-wide HTS data duplications and deletions at the FCGR locus, where CN ranges from zero to four. To date, however, the utility of this approach for genotyping loci at which a greater range of CN exists is untested.

One complex CN variable locus is *CCL3L1*. Copy number variation at *CCL3L1* has been associated with susceptibility to HIV infection (Liu et al., 2010), autoimmune disease (Burns et al., 2005; Mamtani et al., 2008; McKinney et al., 2008) and asthma (Lee et al., 2011). The median CN of *CCL3L1* is 2 in European populations and >2 in other populations (Gonzalez et al., 2005). Evaluation of the role of *CCL3L1* CNV in common disease has been hampered by robustness of methodology, particularly that based on Q-PCR (He et al., 2009; Carpenter et al., 2011). Similarly, CNV within the beta-defensin locus on chromosome 8, which includes the *DEFB4* and *DEFB103A* genes that vary in CN *en bloc* (a range of two to nine copies) (Groth et al., 2008), has been associated with various infectious and inflammatory phenotypes (Bentley et al., 2010; Mehlotra et al., 2012; Zhou et al., 2012). As is the case for *CCL3L1*, however, interpretation of these data, and further progress in studying the role of beta-defensin CN in medical phenotypes, is hampered by the difficulty in accurately measuring CN in large numbers of samples (Aldhous et al., 2010). Paralog ratio test (PRT) copy number data are available at both *CCL3L1* and the beta-defensin locus (Armour et al., 2007; Walker et al., 2009), and thus provide an important resource for developing and validating new HTS read depth-based copy number assignment approaches. The PRT uses multiple probes to compare CN to an invariant paralog and is currently regarded as the gold-standard method for measuring CN at complex loci (McKinney and Merriman, 2012).

Here we describe the development of CNVrd2 as an extension of the CNVrd methodology, and demonstrate the application of this new approach by assigning copy number at the development locus *CCL3L1*, and test locus *DEFB103A*, both of which have previously had CN measured using the PRT.

## Subjects and methods

### HTS and microarray data used

The genomic coordinates (hg19) used in this research were derived from the NCBI (http://www.ncbi.nlm.nih.gov/) and were *CCL3* (chr17:34415602-34417506), *CCL3L1* (chr17:34623842-34625730), *CCL3L3* (chr17:34623842-34625730), *CCL4* (chr17:34431220-34433014), *CCL4L1* (chr17:34538468-34540275), *DEFB103A* (chr8:7738726-7740105) and *DEFB103B* (chr8:7286410-7287682). Data were downloaded from the European Bioinformatics Institute (EBI) FTP server (ftp://ftp.1000genomes.ebi.ac.uk/vol1/ftp/data/ and ftp://ftp.1000genomes.ebi.ac.uk/vol1/ftp/phase1/data/). For *CCL3L1*, 2535 samples (Table 1) downloaded from 26 populations on February 10, 2014 were used. These had an average coverage of 2.8–40.6× over a 2 Mb region around the *CCL3L1* locus. For *DEFB103A*, 2535 samples (Table 2) downloaded on August 13, 2013 were used. These had an average coverage of 2.8–30.9× (median 6.9×) over the 2 Mb region around the *DEFB103A* locus. Only samples sequenced on Illumina platforms were used, so as to avoid cross platform variability. BWA-aligned data were available in BAM format (Li et al., 2009).

**Table 1:** ***CCL3L1* copy-number assignments by CNVrd2**.

		**Copy number**										**Total samples**
		**0**	**1**	**2**	**3**	**4**	**5**	**6**	**7**	**8**	**≥9**	
African ancestry	ACB	0 (0%)	1 (1%)	13 (13.5%)	19 (19.8%)	35 (36.5%)	13 (13.5%)	9 (9.4%)	4 (4.2%)	2 (2.1%)	0 (0%)	96
	ASW	0 (0%)	3 (4.5%)	11 (16.7%)	14 (21.2%)	17 (25.8%)	12 (18.2%)	6 (9.1%)	2 (3%)	1 (1.5%)	0 (0%)	66
	ESN	0 (0%)	0 (0%)	6 (6.1%)	22 (22.2%)	24 (24.2%)	20 (20.2%)	15 (15.2%)	10 (10.1%)	0 (0%)	2 (2%)	99
	GWD	0 (0%)	1 (0.9%)	10 (8.8%)	35 (31%)	27 (23.9%)	21 (18.6%)	10 (8.8%)	7 (6.2%)	2 (1.8%)	0 (0%)	113
	LWK	0 (0%)	2 (2%)	7 (6.9%)	24 (23.8%)	31 (30.7%)	17 (16.8%)	9 (8.9%)	7 (6.9%)	2 (2%)	2 (2%)	101
	MSL	0 (0%)	2 (2.4%)	7 (8.2%)	17 (20%)	33 (38.8%)	15 (17.6%)	5 (5.9%)	5 (5.9%)	0 (0%)	1 (1.2%)	85
	YRI	0 (0%)	1 (0.9%)	14 (12.8%)	19 (17.4%)	30 (27.5%)	25 (22.9%)	8 (7.3%)	9 (8.3%)	2 (1.8%)	1 (0.9%)	109
Americas	CLM	6 (6.4%)	8 (8.5%)	29 (30.9%)	35 (37.2%)	11 (11.7%)	5 (5.3%)	0 (0%)	0 (0%)	0 (0%)	0 (0%)	94
	MXL	0 (0%)	8 (11.9%)	15 (22.4%)	26 (38.8%)	10 (14.9%)	7 (10.4%)	1 (1.5%)	0 (0%)	0 (0%)	0 (0%)	67
	PEL	0 (0%)	7 (8.1%)	9 (10.5%)	45 (52.3%)	21 (24.4%)	3 (3.5%)	0 (0%)	0 (0%)	1 (1.2%)	0 (0%)	86
	PUR	4 (3.8%)	18 (17.1%)	31 (29.5%)	34 (32.4%)	11 (10.5%)	4 (3.8%)	1 (1%)	0 (0%)	2 (1.9%)	0 (0%)	105
East Asian ancestry	CDX	1 (1%)	12 (12.1%)	23 (23.2%)	46 (46.5%)	8 (8.1%)	7 (7.1%)	0 (0%)	1 (1%)	1 (1%)	0 (0%)	99
	CHB	1 (1%)	3 (2.9%)	11 (10.7%)	30 (29.1%)	20 (19.4%)	21 (20.4%)	7 (6.8%)	5 (4.9%)	4 (3.9%)	1 (1%)	103
	CHS	1 (0.9%)	8 (7.4%)	16 (14.8%)	38 (35.2%)	12 (11.1%)	20 (18.5%)	9 (8.3%)	0 (0%)	3 (2.8%)	1 (0.9%)	108
	JPT	0 (0%)	4 (3.8%)	12 (11.5%)	40 (38.5%)	11 (10.6%)	19 (18.3%)	7 (6.7%)	10 (9.6%)	0 (0%)	1 (1%)	104
	KHV	3 (3%)	10 (9.9%)	18 (17.8%)	35 (34.7%)	16 (15.8%)	11 (10.9%)	4 (4%)	4 (4%)	0 (0%)	0 (0%)	101
European ancestry	CEU	2 (2%)	26 (26.3%)	51 (51.5%)	16 (16.2%)	4 (4%)	0 (0%)	0 (0%)	0 (0%)	0 (0%)	0 (0%)	99
	FIN	2 (2%)	23 (23.2%)	36 (36.4%)	33 (33.3%)	5 (5.1%)	0 (0%)	0 (0%)	0 (0%)	0 (0%)	0 (0%)	99
	GBR	1 (1.1%)	21 (22.8%)	50 (54.3%)	16 (17.4%)	4 (4.3%)	0 (0%)	0 (0%)	0 (0%)	0 (0%)	0 (0%)	92
	IBS	3 (2.8%)	33 (30.8%)	45 (42.1%)	22 (20.6%)	4 (3.7%)	0 (0%)	0 (0%)	0 (0%)	0 (0%)	0 (0%)	107
	TSI	7 (6.5%)	34 (31.5%)	38 (35.2%)	23 (21.3%)	5 (4.6%)	1 (0.9%)	0 (0%)	0 (0%)	0 (0%)	0 (0%)	108
South Asian ancestry	BEB	2 (2.3%)	9 (10.5%)	35 (40.7%)	24 (27.9%)	10 (11.6%)	3 (3.5%)	2 (2.3%)	1 (1.2%)	0 (0%)	0 (0%)	86
	GIH	2 (1.9%)	11 (10.4%)	51 (48.1%)	35 (33%)	6 (5.7%)	1 (0.9%)	0 (0%)	0 (0%)	0 (0%)	0 (0%)	106
	ITU	3 (2.9%)	16 (15.5%)	43 (41.7%)	30 (29.1%)	8 (7.8%)	3 (2.9%)	0 (0%)	0 (0%)	0 (0%)	0 (0%)	103
	PJL	4 (4.2%)	12 (12.5%)	39 (40.6%)	30 (31.2%)	6 (6.2%)	5 (5.2%)	0 (0%)	0 (0%)	0 (0%)	0 (0%)	96
	STU	1 (1%)	18 (17.5%)	40 (38.8%)	32 (31.1%)	7 (6.8%)	4 (3.9%)	1 (1%)	0 (0%)	0 (0%)	0 (0%)	103

*European ancestry: Northern and Western European ancestry residing in Utah, USA (CEU), Toscani in Italia (TSI), British from England and Scotland (GBR), Finnish from Finland (FIN), Iberian populations in Spain (IBS); African ancestry: African Ancestry in Southwest US (ASW), African Caribbean in Barbados (ACB), Yoruba in Ibadan, Nigeria (YRI), Luhya in Webuye, Kenya (LWK), Gambian in Western Division, The Gambia (GWD), Mende in Sierra Leone (MSL), Esan in Nigeria (ESN); East Asian ancestry: Han Chinese in Beijing, China (CHB), Japanese in Toyko, Japan (JPT), Han Chinese South (CHS), Kinh in Ho Chi Minh City, Vietnam (KHV), Chinese Dai in Xishuangbanna (CDX); Americas: Mexican Ancestry in Los Angeles (MXL), Puerto Rican in Puerto Rico (PUR), Colombian in Medellin, Colombia (CLM), Peruvian in Lima, Peru (PEL); South Asian ancestry: Gujarati Indian in Houston, TX (GIH), Bengali in Bangladesh (BEB), Indian Telegu in the UK (ITU), Punjabi in Lahore (PJL), Sri Lankan Tamil in the UK (STU)*.

**Table 2:** ***DEFB103A* copy-number assignments by CNVrd2**.

		**Copy number**								**Total sample**
		**2**	**3**	**4**	**5**	**6**	**7**	**8**	**≥9**	
African ancestry	ACB	3 (3.1%)	11 (11.5%)	28 (29.2%)	27 (28.1%)	15 (15.6%)	8 (8.3%)	3 (3.1%)	1 (1%)	96
	ASW	0 (0%)	8 (12.1%)	20 (30.3%)	17 (25.8%)	13 (19.7%)	4 (6.1%)	3 (4.5%)	1 (1.5%)	66
	ESN	0 (0%)	19 (19.2%)	25 (25.3%)	24 (24.2%)	17 (17.2%)	10 (10.1%)	3 (3%)	1 (1%)	99
	GWD	0 (0%)	9 (8%)	30 (26.5%)	39 (34.5%)	19 (16.8%)	12 (10.6%)	3 (2.7%)	1 (0.9%)	113
	LWK	0 (0%)	15 (14.9%)	28 (27.7%)	21 (20.8%)	19 (18.8%)	15 (14.9%)	3 (3%)	0 (0%)	101
	MSL	1 (1.2%)	6 (7.1%)	28 (32.9%)	30 (35.3%)	13 (15.3%)	3 (3.5%)	2 (2.4%)	2 (2.4%)	85
	YRI	2 (1.8%)	14 (12.8%)	34 (31.2%)	29 (26.6%)	17 (15.6%)	7 (6.4%)	4 (3.7%)	2 (1.8%)	109
Americas	CLM	2 (2.1%)	15 (16%)	46 (48.9%)	24 (25.5%)	5 (5.3%)	2 (2.1%)	0 (0%)	0 (0%)	94
	MXL	1 (1.5%)	10 (14.9%)	26 (38.8%)	23 (34.3%)	4 (6%)	2 (3%)	1 (1.5%)	0 (0%)	67
	PEL	5 (5.8%)	16 (18.6%)	35 (40.7%)	22 (25.6%)	6 (7%)	2 (2.3%)	0 (0%)	0 (0%)	86
	PUR	3 (2.9%)	24 (22.9%)	41 (39%)	26 (24.8%)	8 (7.6%)	0 (0%)	3 (2.9%)	0 (0%)	105
East Asian ancestry	CDX	2 (2%)	17 (17.2%)	38 (38.4%)	29 (29.3%)	10 (10.1%)	1 (1%)	1 (1%)	1 (1%)	99
	CHB	3 (2.9%)	22 (21.4%)	45 (43.7%)	21 (20.4%)	11 (10.7%)	1 (1%)	0 (0%)	0 (0%)	103
	CHS	3 (2.8%)	21 (19.4%)	45 (41.7%)	26 (24.1%)	9 (8.3%)	3 (2.8%)	1 (0.9%)	0 (0%)	108
	JPT	5 (4.8%)	18 (17.3%)	43 (41.3%)	24 (23.1%)	9 (8.7%)	4 (3.8%)	1 (1%)	0 (0%)	104
	KHV	1 (1%)	21 (20.8%)	35 (34.7%)	28 (27.7%)	10 (9.9%)	4 (4%)	1 (1%)	1 (1%)	101
European ancestry	CEU	3 (3%)	9 (9.1%)	49 (49.5%)	24 (24.2%)	10 (10.1%)	2 (2%)	2 (2%)	0 (0%)	99
	FIN	1 (1%)	11 (11.1%)	46 (46.5%)	30 (30.3%)	10 (10.1%)	1 (1%)	0 (0%)	0 (0%)	99
	GBR	1 (1.1%)	9 (9.8%)	38 (41.3%)	30 (32.6%)	11 (12%)	3 (3.3%)	0 (0%)	0 (0%)	92
	IBS	6 (5.6%)	19 (17.8%)	41 (38.3%)	31 (29%)	8 (7.5%)	2 (1.9%)	0 (0%)	0 (0%)	107
	TSI	3 (2.8%)	22 (20.4%)	47 (43.5%)	22 (20.4%)	11 (10.2%)	3 (2.8%)	0 (0%)	0 (0%)	108
South Asian ancestry	BEB	5 (5.8%)	14 (16.3%)	44 (51.2%)	11 (12.8%)	11 (12.8%)	1 (1.2%)	0 (0%)	0 (0%)	86
	GIH	4 (3.8%)	15 (14.2%)	44 (41.5%)	27 (25.5%)	12 (11.3%)	4 (3.8%)	0 (0%)	0 (0%)	106
	ITU	4 (3.9%)	24 (23.3%)	41 (39.8%)	18 (17.5%)	14 (13.6%)	2 (1.9%)	0 (0%)	0 (0%)	103
	PJL	3 (3.1%)	17 (17.7%)	37 (38.5%)	23 (24%)	13 (13.5%)	2 (2.1%)	1 (1%)	0 (0%)	96
	STU	3 (2.9%)	18 (17.5%)	51 (49.5%)	22 (21.4%)	8 (7.8%)	1 (1%)	0 (0%)	0 (0%)	103

Refer to Table 1 legend for key to population abbreviations.

### The workflow of the pipeline

**Figure 1:**
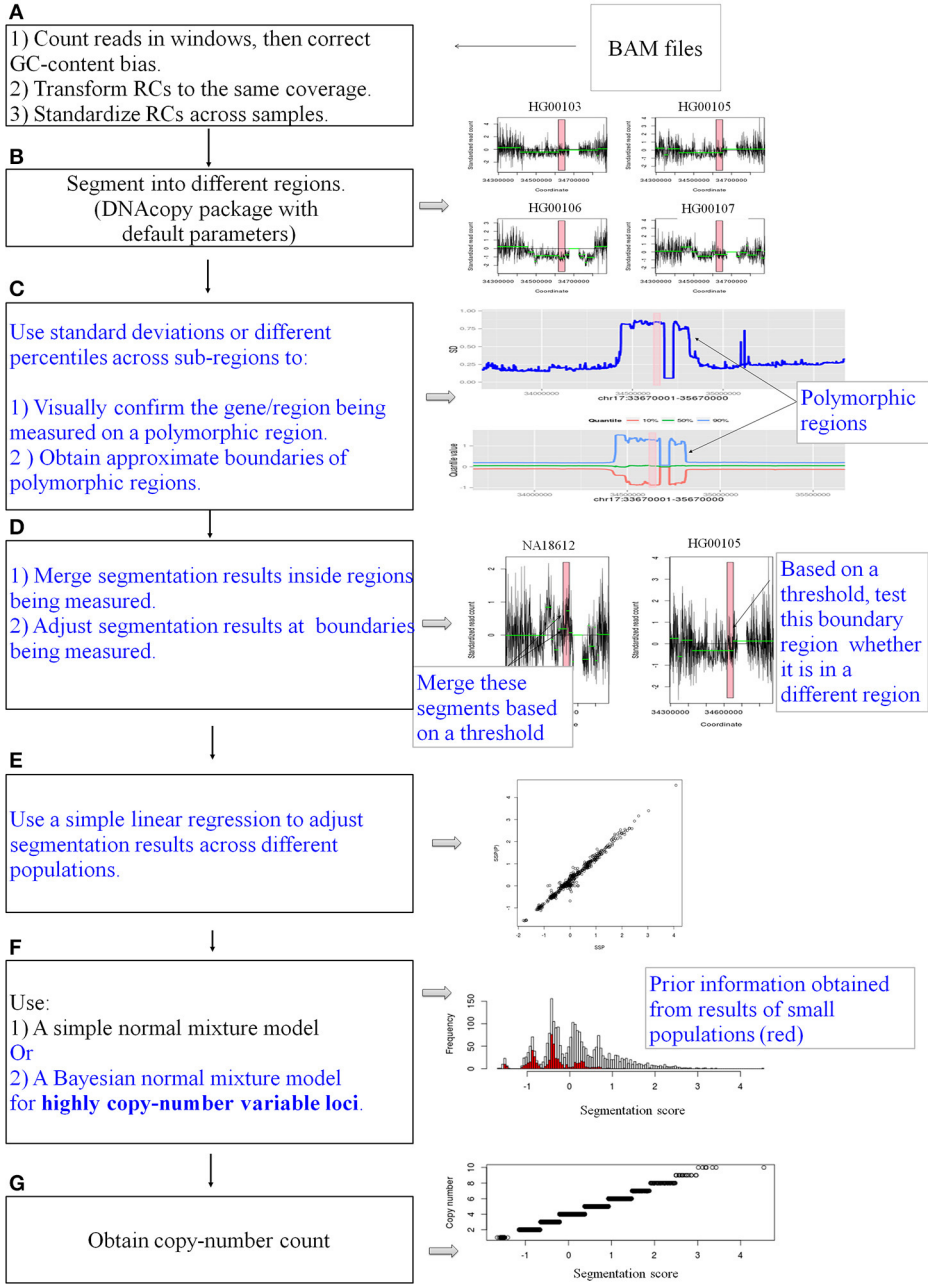
**A schematic diagram of the pipeline**. CNVrd2 is a modified version of CNVrd. CNVrd2 is identical to CNVrd at the counting, transforming, standardizing and segmenting steps (**A,B,G**: black text). However, CNVrd2 has additional steps: identification of polymorphic regions **(C)**, merging sub-regions inside genes/regions being measured and testing boundary regions **(D)**, using a simple linear regression model to adjust segmentation scores between populations **(E)** and a Bayesian normal mixture to cluster segmentation scores of highly CN variable regions into different groups **(F)**. These new steps are in blue text.

The entire pipeline is represented in Figure 1. The pipeline is used to measure CN for a specific gene or locus. Four main steps are described below.

### Pipeline workflow: identification of approximate boundaries of measured regions

Samples were segmented as previously described for the CNVrd procedure (Nguyen et al., 2013). The results of the segmentation process were used to obtain approximate boundaries of copy-number variable regions encompassing the gene being measured. CNVrd2 used different percentiles and standard deviations of segmentation results to visually obtain information of CNVs at loci. Then, standard deviations were used to obtain approximate boundaries as follows (Step C, Figure 1).

To identify approximate boundaries of putative polymorphic regions (Step C, Figure 1), the coordinates of regions having similar segmentation results for each sample were obtained from the segmentation process applied to the large 1-2 Mb regions across all samples. Next, all these coordinates were combined to generate sub-regions, and segmentation results generated by the original segmentation analysis for each sample were obtained. Finally, the standard deviations and various percentiles of segmentation results of each sub-region across the 2535 samples were calculated and plotted to visually confirm CNV. Windows exhibiting large standard deviations were used to identify approximate positions of putative regions being assigned CN.

### Pipeline workflow: obtaining segmentation scores for individual samples at the measured locus

This step was similar to our previous pipeline (CNVrd) but with some modifications (Step D, Figure 1) added to obtain more reliable segmentation scores. If the entirety of the gene of interest was placed in a single segment then the segmentation score for the gene was the segmentation score for that region. If the gene was split into multiple segments, each having the same sign then the segmentation score for the gene was calculated as the length-weighted average across the segments (NA18612 for example in Step D, Figure 1). Otherwise, if a segment at the boundary of the gene had a different sign from other sub-regions of the gene (HG00254 for example) and the boundary segment's length was less than a specific threshold (here the threshold was set to half the window size) then the z-score for the boundary segment was calculated, where the z-score is defined as the value of the standardized observed read-count ratio (Nguyen et al., 2013). If the z-score had the same sign as the segmentation scores from other sub-regions of the gene, then the segmentation score for the gene was again calculated as the length-weighted average across the other segments. For all other situations the gene of interest was assigned a segmentation score of zero, reflecting the population average at that locus.

### Pipeline workflow: adjustment of the segmentation process for multiple populations

**Figure 2:**
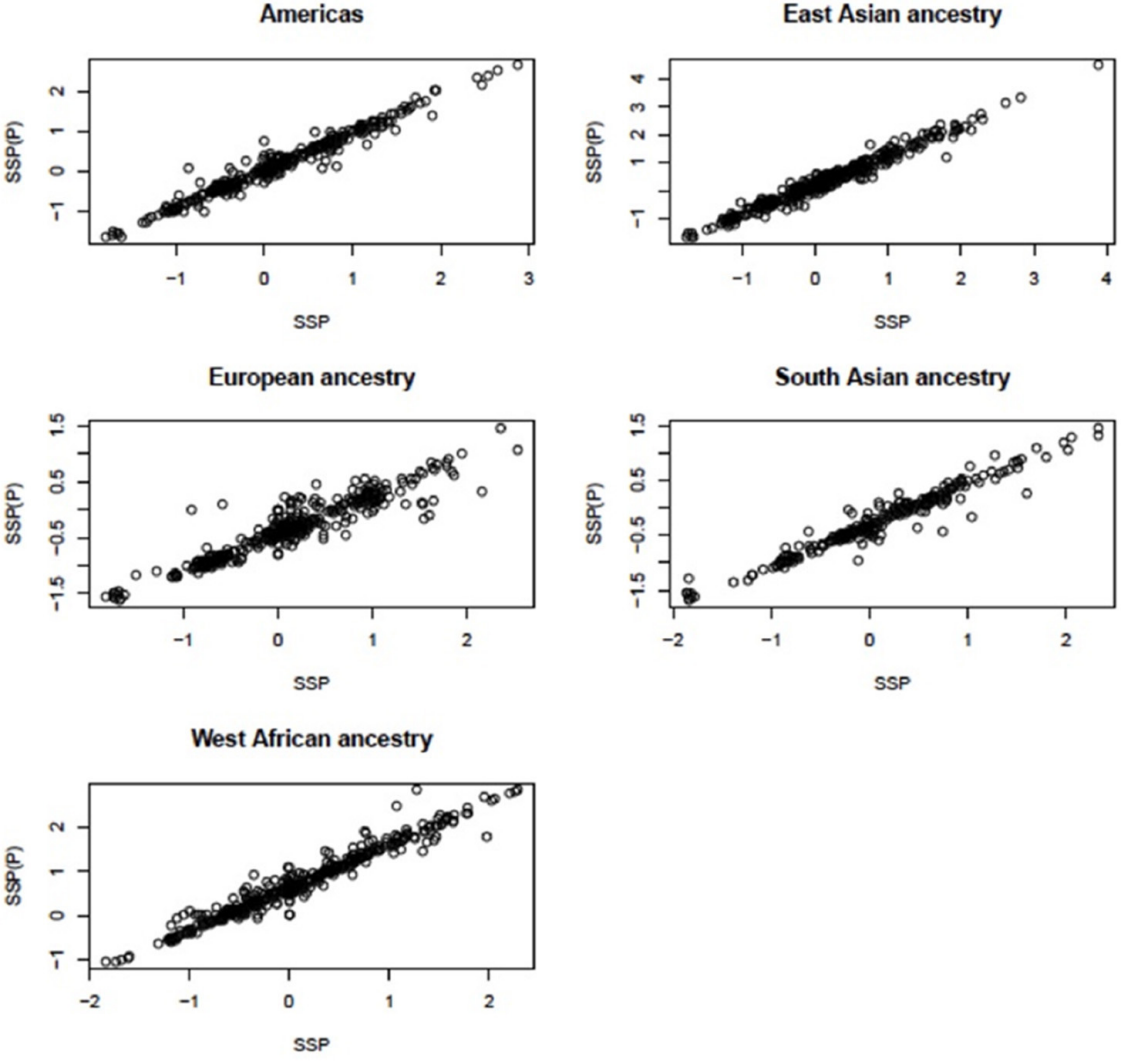
**Linear relationship between the segmentation scores called for single populations and for all populations at *CCL3L1***. SSP, the segmentation scores of a single large population; SSP(_P_), the segmentation score of pooled populations.

The pipeline was applied to each of the five major populations (European, East Asian, West African, South Asian, and Americas) to obtain segmentation scores (SSP: the segmentation scores for a single major population). This identified the scores in each of the large populations, but did not allow comparisons between the populations. Therefore, we ran the segmentation process for all samples and obtained segmentation scores for samples in all populations [SSP_(P)_: the segmentation scores for pooled populations]. When we pooled samples and ran the pipeline, if the gene being analyzed was not segmented into a single region for a particular sample, then that sample was assigned a segmentation score of zero. For example, the median *CCL3L1* CN for European-ancestry sample sets was less than other populations, therefore the majority of segmentation results and z-scores (where needed—see above) were less than zero. If the *CCL3L1* gene region of a European-ancestry sample was segmented into two sub-regions, both having negative signs in the all-population analysis, then this could either be recapitulated in the single-population analysis (implying they were within the same CN region) or they could be sub-regions that could span the population average. Thus, they could have one negative and one positive sign (implying they were within two distinct CN regions). In this case, however, the pipeline only recognized that sub-regions shared the same sign, and the two regions would therefore be considered as one region and would be merged. To improve this, for each of the five major populations, we fitted a linear regression line with SSP and SSP_(P)_ as independent and dependent variables respectively (data for *CCL3L1* are shown in Figure 2). This step aimed to reduce errors when performing segmentation within single major populations or pooled populations. The fitted mean values of the five single linear regression models were used as the final per sample segmentation scores for these five populations. Segmentation scores were transformed and standardized, and a normal mixture model was used to cluster the scores into CN genotype groups.

### Pipeline workflow: assigning copy-number counts

A normal mixture model was used to cluster final segmentation scores into different groups. If clear clusters were observed then the Expectation Maximization (EM) algorithm (Dempster et al., 1977) was used in the clustering process. If data were complicated (e.g., loci with high copy-number variants such as *CCL3L1* and *DEFB103A*) then a Bayesian clustering approach was used (Step F, Figure 1; details of the analysis process for *CCL3L1* and *DEFB103A* are described below).

### Identification of window size parameters for CNVrd2 using test loci

One of the new innovations in CNVrd2 was the merging of segmentation results inside regions being measured if these regions were divided into different parts (Step D, Figure 1). We merged these parts if their signal values were above (below) a threshold (namely “testThreshold2Merge” in the CNVrd2 package) for duplicated events (deleted events). Another parameter was the length of a boundary region, the default for which was set to “half window size.” Real data sets were used for investigation of the parameters of CNVrd2. In order to make reliable data sets we intersected the CNV results of two sets of data (Conrad et al., 2010; Campbell et al., 2011). From these intersecting data we chose eight loci whose copy-number assignments were ≥45% identical and whose lengths were in different ranges (~3, ~5, ~8, ~20, ~24, ~45, ~60, ~100 kb). The coordinates and CN status of the 8 loci are described in Table 3. We only retained samples having identical results between the methods. We downloaded BAM files of the 1 Mb regions around these loci and let CNVrd2 automatically assign CN for the loci (CN groups of these loci were known in advance from the micro-array based results, Table 3).

**Table 3:** **Eight loci which were obtained from the intersection of results of Conrad et al. (2010) and Campbell et al. (2011) were used to obtain parameters for CNVrd2 and to compare between CNVrd2 and CNVrd**.

**Locus**	**Region downloaded**	**Sample size^a^**	**CN^b^**
chr7:141769627-141793931	7:141000000-142000000	258	0 (3; 1.2) 1 (44; 17.1) 2 (208; 80.6) 3 (3; 1.2)
chr3:162514938-162619146	3:162000000-163000000	136	0 (93; 68.4) 1 (28; 20.6) 2 (15; 11.0)
chr17:44212815-44270230	17:43500000-44500000	252	2 (195; 77.4) 3 (38; 15.1) 4 (19; 7.5)
chr1:110222301-110242933	1:109500000-110500000	251	2 (106; 42.2) 3 (102; 40.6) 4 (43; 17.1)
chr17:44212815-44270230	17:43500000-44500000	252	2 (195; 77.4) 3 (38; 15.1) 4 (19; 7.5)
chr2:79331533-79339762	2:79000000-80000000	231	2 (218; 94.4) 3 (13; 5.6)
chr16:72109587-72112297	16:71500000-72500000	251	2 (226; 90.0) 3 (25; 10.0)
chr3:26434104-26439360	3:26000000-27000000	259	0 (3; 1.2) 1 (14; 5.4) 2 (240; 92.7) 3 (2; 0.8)

a *These samples result from the intersection of the samples of Conrad et al. and Campbell et al. (Conrad et al., 2010; Campbell et al., 2011) and the 1000 Genomes Project*.

b *In parentheses are the number and percentage of samples with the specified CN, as obtained from Conrad et al. (2010) and Campbell et al. (2011)*.

### Comparison with CNVrd

We compared the new pipeline with our previous pipeline at the eight loci mentioned above (Table 3) and the eleven loci previously measured in Nguyen et al. (2013). For the eight loci, both pipelines were run and the percent concordances were calculated. For CNVrd2, we used different window sizes (0.1, 0.2, 0.5, 1, 2, 5, 10, 20, and 50 kb) and different values (0.15, 0.25, 0.35, and 0.45) of testThreshold2Merge. For CNVrd, we only used different windows (0.1, 0.2, 0.5, 1, 2, 5, 10, 20, and 50 kb) because the merging process was not implemented in CNVrd. For the eleven loci, we used a “testThreshold2Merge” value of 0.35 and the same window size as CNVrd [*FCGR3A* and *FCBR3B* (1000 bp), *RHD* (2000 bp), *UGT2B17* (500 bp), *GSTT1* (1000 bp), *IGLL3P* (1000 bp), *SMN2* (1000 bp), *GSTM1* (200 bp), *CFHR1* (500 bp), *CNTNAP3* (2000 bp) and *IGLL5* (1000 bp)].

### Application of CNVrd2 to two complex loci: CCL3L1 and DEFB103A

Windows of 500 and 1000 bp were used to analyze *CCL3L1* and *DEFB103A*, respectively. Steps described above were used to obtain putative boundaries of *CCL3L1/DEFB103A* gene-containing regions and final segmentation scores for the two loci. For each locus, one population having clear clusters of segmentation scores was used to obtain prior information for all populations: the European and South Asian population for *CCL3L1* and *DEFB103A*, respectively. This approach was implemented using the EM algorithm (Dempster et al., 1977). The results for the European and South Asian populations (means and variances of groups and the distances between the means of the groups) were used to provide prior information for a Bayesian normal mixture model that was applied to the other populations. This was done to improve model stability and fit for populations exhibiting high levels of CN polymorphism. The Markov chain Monte Carlo sampling method implemented in the rjags package (Plummer, 2011) was used to obtain posterior estimates for each normal mixture model parameter—this allows control of parameters using prior information. An adaptive phase of 100 iterations was run, followed by a burn-in period of 1000 iterations. Next, we ran 20,000 iterations and calculated the means, standard deviations, and proportions of the mixture components from 20% of the iterations. Convergence was assessed by using Heidelberger and Welch's stationarity and half-width test implemented in the coda package (Plummer et al., 2006).

### Comparison with other methods: CCL3L1

**Figure 3:**
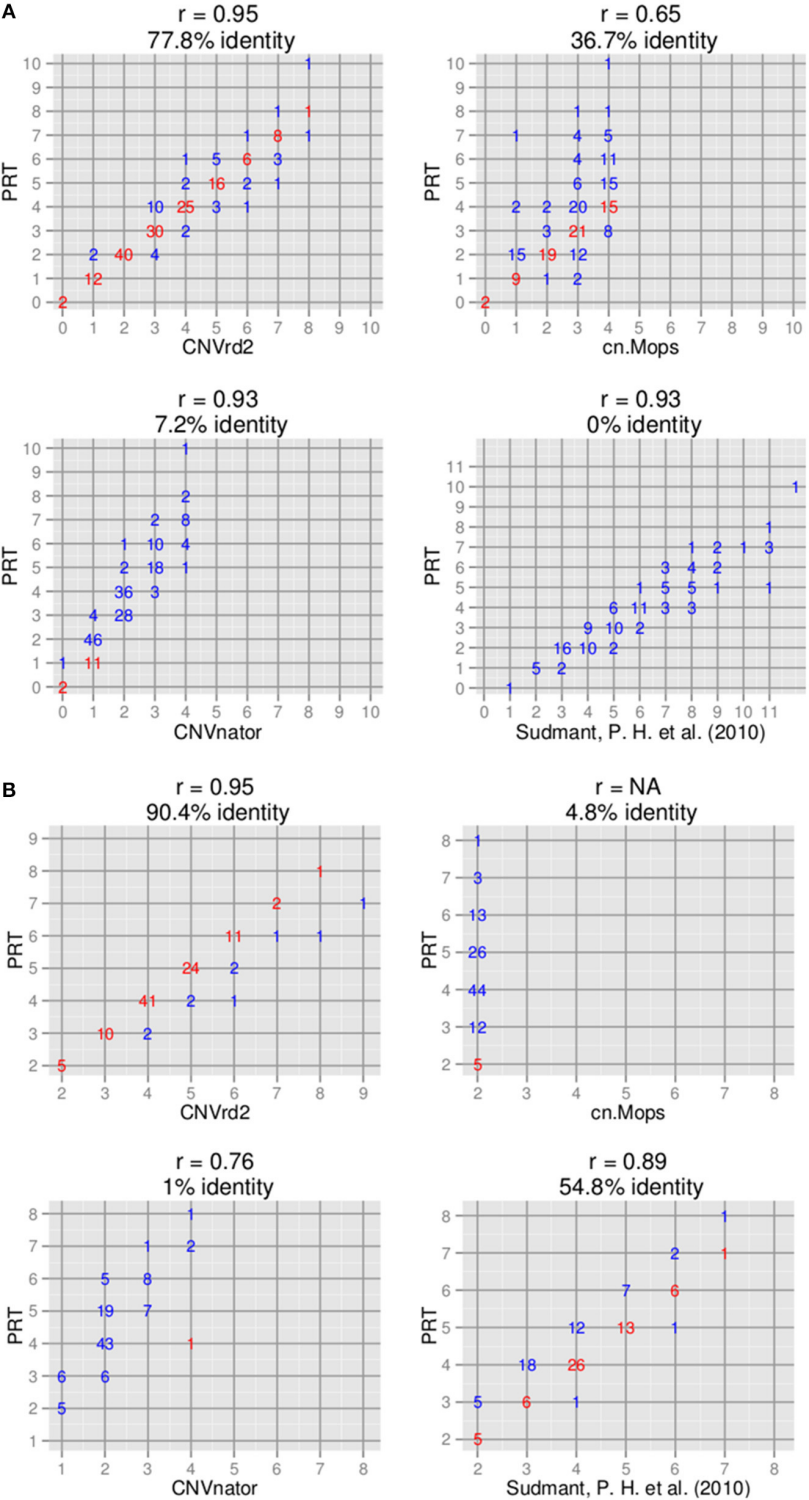
**Comparison of copy number assignments of high-throughput sequencing-based with PRT-based methods**. **(A)** *CCL3L1* on 180 samples [only 111 samples measured by Sudmant et al. (2010) overlapped]. **(B)** *DEFB103A* on 104 samples.

Copy number assignments at *CCL3L1* by cn.Mops (Klambauer et al., 2012), CNVnator (Abyzov et al., 2011) and Sudmant et al. (2010) were compared to assignments of CNVrd2 on the 180 samples measured by the modified PRT. To run CNVnator, we downloaded all of the chromosome 17 data for the 180 samples. CNVnator was applied as previously described (Nguyen et al., 2013). cn.Mops uses read-count information of single samples and multiple samples to detect CN variable regions and uses a Poisson mixture model to automatically infer CN. We used CNVrd2 to obtain matrices of read counts in the 2 Mb region (chr17:33670000-34670000) in eight different scenarios: constant windows of 25000, 20000, 10000, 5000, 2000, 1000, 500, and 200 bp with default values for other parameters. The 5000 bp window for cn.Mops had the highest proportion of samples having *CN* > 3 and the highest concordance with other methods and was used in the Figure 3 comparison. The Sudmant et al. (2010) read count-based method utilized all possible mapping locations of a read combined with singly unique nucleotide positions to measure CN for 169 samples from the 1000 Genomes Project at different loci, including the *CCL3L1* gene. The *CCL3L1* gene results were validated by using Q-PCR based assays with high correlation being observed (*r* = 0.95) (Sudmant et al., 2010). 111 of the 169 samples overlapped with the samples analyzed here using CNVrd2, and by Carpenter et al. (2011) using the PRT-based methods. The coordinates of *CCL3L1* in the Sudmant et al. (2010) data were chr17:34623842-34625730 (hg19).

### Comparison with other methods: DEFB103A

The data sets of Sudmant et al. (2010) and Hardwick et al. (2011) were intersected and 104 samples were obtained that overlapped with the 2535 1000 Genomes samples we analyzed using CNVrd2. The coordinates of the DEFB103A gene in the Sudmant et al. (2010) data were chr8:7738913-7740180 (hg19). For CNVnator, we downloaded all of the chromosome 8 data for the 104 samples and used a similar approach as for *CCL3L1*. For cn.Mops, we used CNVrd2 to obtain matrices of read counts in the 2 Mb region (chr8:6500000-8500000) for 2535 samples in eight different scenarios: constant windows of 25000, 20000, 10000, 5000, 2000, 1000, 500, and 200 bp with default values for other parameters. The 10000 bp window was the only window length to call CN (six samples assigned *CN* = 3, the remainder assigned *CN* = 2) and was used in the Figure 3 comparison.

LiftOver (Meyer et al., 2013) on the UCSC Genome browser was used to convert coordinates of microarray data and the Sudmant et al. (2010) data from Human Reference Genome hg18 to Human Reference Genome hg19.

## Results

### Data

**Figure 4:**
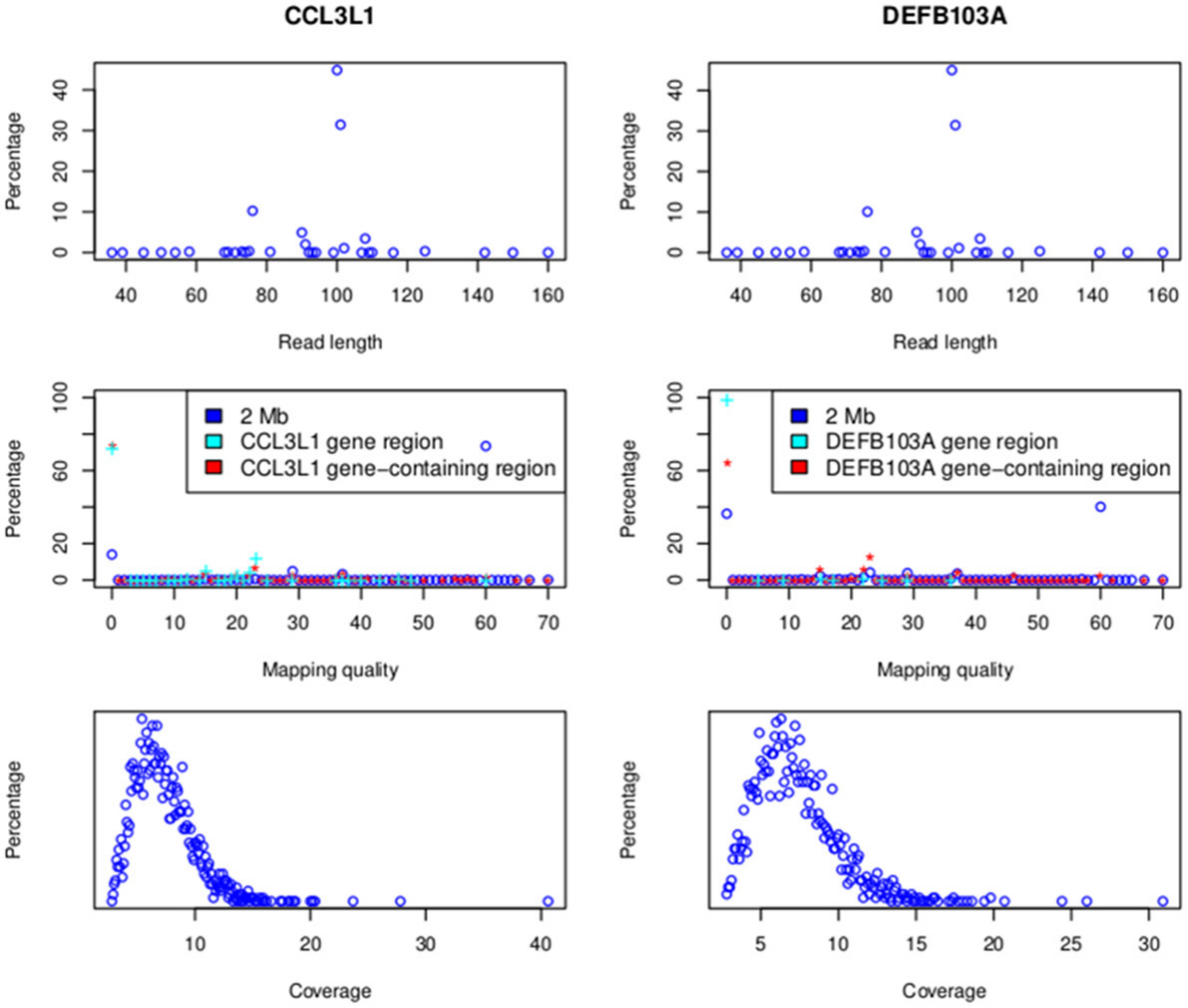
**Read lengths and mapping qualities (top), mapping qualities (middle) and average read depth (bottom)**. Data for the 2 MB *CCL3L1* region are on the left and the 2 Mb *DEFB103A* region on the right.

Alignment results are presented in Figure 4. At *CCL3L1* a total of 395,078,047 reads across all samples were aligned to a 2 Mb region around the gene (Chr17:33670000-35670000). These reads had lengths ranging from 36 to 160 bp (median of 91.5 bp and the highest frequency, 44.9%, was 100 bp), and mapping qualities from 0 to 70 (median of 33.5 and the highest frequency, 73.4%, was 60). The majority of reads (73.5% and 72.5%) aligning to the CCL3L1-containing region (chr17:34617501-3465201) and *CCL3L1* gene (Chr17:34623842-34625730) had a mapping quality of 0, presumably reflecting multiple alignments to the paralogs within the locus.

A total of 386,195,488 reads across all samples were aligned to the 2 Mb region on Chr 8 (Chr8:6500000-8500000) around the defensin genes (Figure 4). These reads had lengths ranging from 36 to 160 bp (median of 91.5 bp and the highest frequency, 45%, was 100 bp), and mapping qualities from 0 to 70 (median of 33.5 and the highest frequency, 40.2%, was 60). The majority of reads (64.1 and 99%, respectively) aligning to the DEFB103A-containing gene (chr8:7641001-7742001) and *DEFB103A* region (Chr8:7738726-7740105) had a mapping quality of 0. The average coverage of 2535 samples on the 2 Mb region was between 2.8 and 30.9× with a median of 6.9× (Figure 4).

### CNVrd2: inferring reliable values for parameters

**Figure 5:**
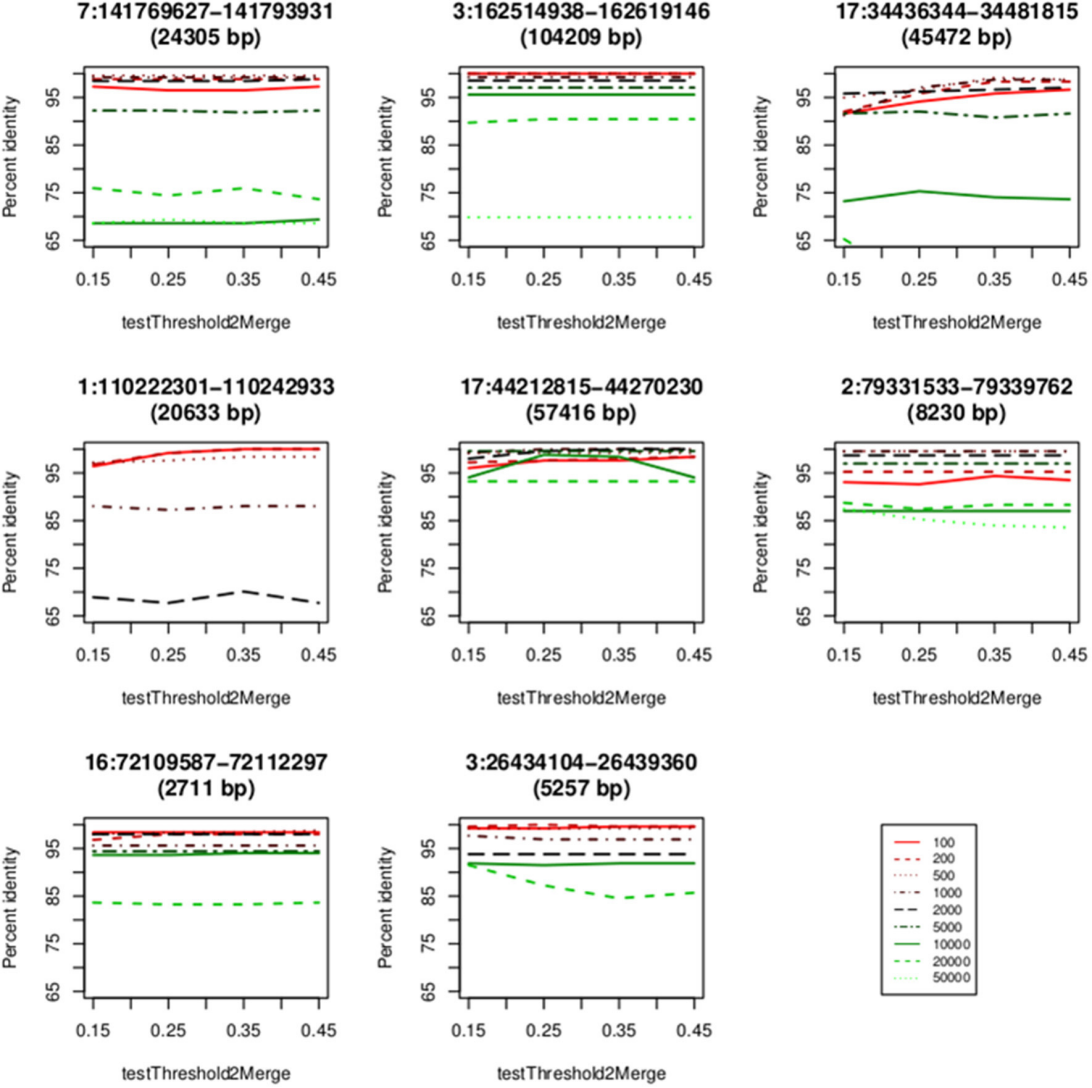
**Concordance between the CNVrd2 and microarray based results**. The x-axis contains the values of testThreshold2Merge (0.15, 0.25, 0.35, 0.45). The y-axis is the percentage of identical results.

We used eight loci derived from the data sets of two microarray approaches (Conrad et al., 2010; Campbell et al., 2011) to obtain parameters of CNVrd2. CNVrd2 had good performance when the window size was 200 or 500 bp and a testThreshold2Merge value of 0.35 was used (Figure 5). A small window size (e.g., 100 bp) was not stable at all loci, possibly because there were multiple low-coverage samples in the 1000 Genomes Project data (e.g., 464/2535 = 18.2% samples having coverage in the *CCL3L1* gene-containing 2 Mb region < 5×, Figure 4). This could lead to some windows having very low read counts, and thus being segmented incorrectly. For example, some windows in normal regions (i.e., *CN* = 2) would be segmented into deleted regions or some windows in duplicated regions would be segmented into normal regions. Similarly, large windows (e.g., 5000 bp) also generated unreliable results (Figure 5).

### CNVrd2: comparison with CNVrd at eight test loci

**Figure 6:**
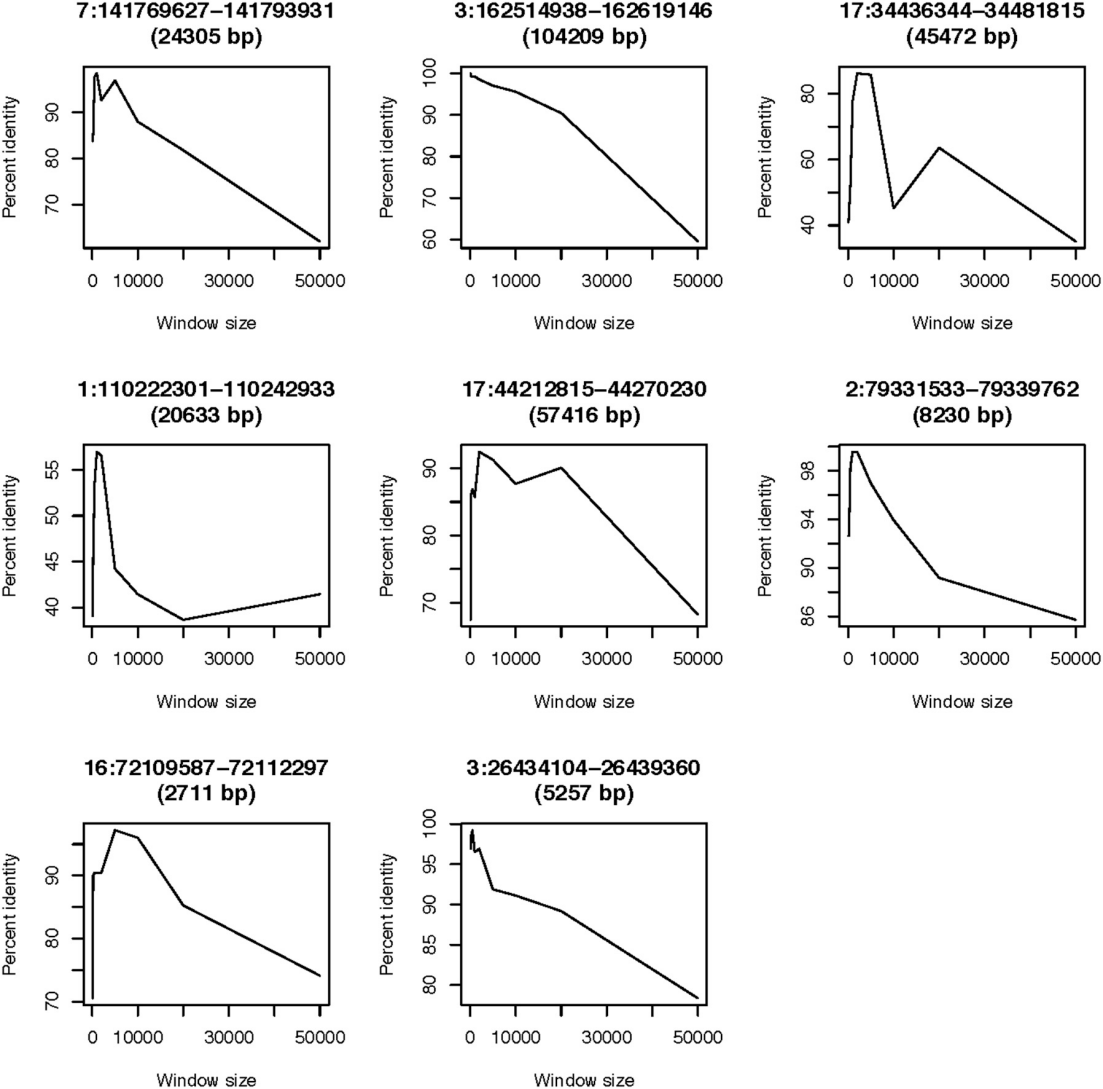
**Concordance between the CNVrd and microarray based results**. Window sizes are shown on the x-axis, while the y-axis shows the percentage of identical results.

Our previous work was focused on loci having low copy-number ranges. Even though this present study is concentrated on loci with *CN* range from zero to >5 *CN*, we also compared with CNVrd (Nguyen et al., 2013) to assess the performance of the modified pipeline, especially in the steps requiring obtaining and adjusting segmentation scores for each sample. Loci used in this comparison had copy number ranging from 0 to 5, and their boundaries were known (Table 3 and Nguyen et al., 2013). Therefore, a simple normal mixture model was used in CNVrd2 to cluster segmentation scores into different groups. CNVrd2 had more stable results than those of CNVrd at almost all window sizes (Figures 5, 6). Merging sub-regions and adjusting boundaries (Step D and E, Figure 1) produced more reliable results for CNVrd2 at two complex regions (chr17:34436344-34481815 and chr1:110222301-110242933). At these two regions, CNVrd showed very low concordance with microarray-based approaches (Figure 6).

### CNVrd2: comparison with the eleven loci used in CNVrd

We also applied CNVrd2 to eleven CNV regions (including *FCGR3A/3B*) to which CN was assigned using CNVrd in our previous work (Nguyen et al., 2013). Better concordances were seen at five loci for CNVrd2 (*CFHR1*: 94.29/91.9%, *RHD*: 99.05/98.57%, *GSTM1*: 95.24/93.81%, *SMN2*: 90.95/88.1%, *UGT2B17*: 99.05/97.14%) and the same concordances were seen at the other four loci (*GSTT1*: 94.76%, *IGLL5*: 95.24%, *CNTNAP3*: 81.43%, *IGLL3P*: 99.52%). For *FCGR3A* and *FCGR3B*, applying CNVrd2 to the same set of 952 samples in our previous work produced slightly higher concordances than for CNVrd when compared with PRT-based *CN* assignments (Hollox et al., 2009) (*FCGR3A*: 84.2/82% and *FCGR3B*:83.5/82.7% for CNVrd2 and CNVrd, respectively).

### Application of CNVrd2 to two complex loci; CCL3L1 and DEFB103A

Information on read length, mapping quality, read depth and read count ratios is presented in Subjects and Methods (“HTS and microarray data used”) and in Figure 4.

Based on the results of the eight test loci (above, Figures 5, 6), the BAM files were processed using 500 bp windows for *CCL3L1* and 1000 bp windows for *DEFB103A*, and a testThreshold2Merge = 0.35. 1000 bp windows were chosen for *DEFB103A* because there was higher correlation between segmentation scores of *DEFB103A* and *DEFB103B* than for 500 bp windows (*r* = 0.80 and 0.78, respectively). Owing to the small length of each gene (1888 bp for *CCL3L1* and 1379 bp for *DEFB103A*) larger window sizes were not investigated as this could result in not detecting breakpoints nearby or inside the genes.

### Polymorphism of the CCL3L1 locus

**Figure 7:**
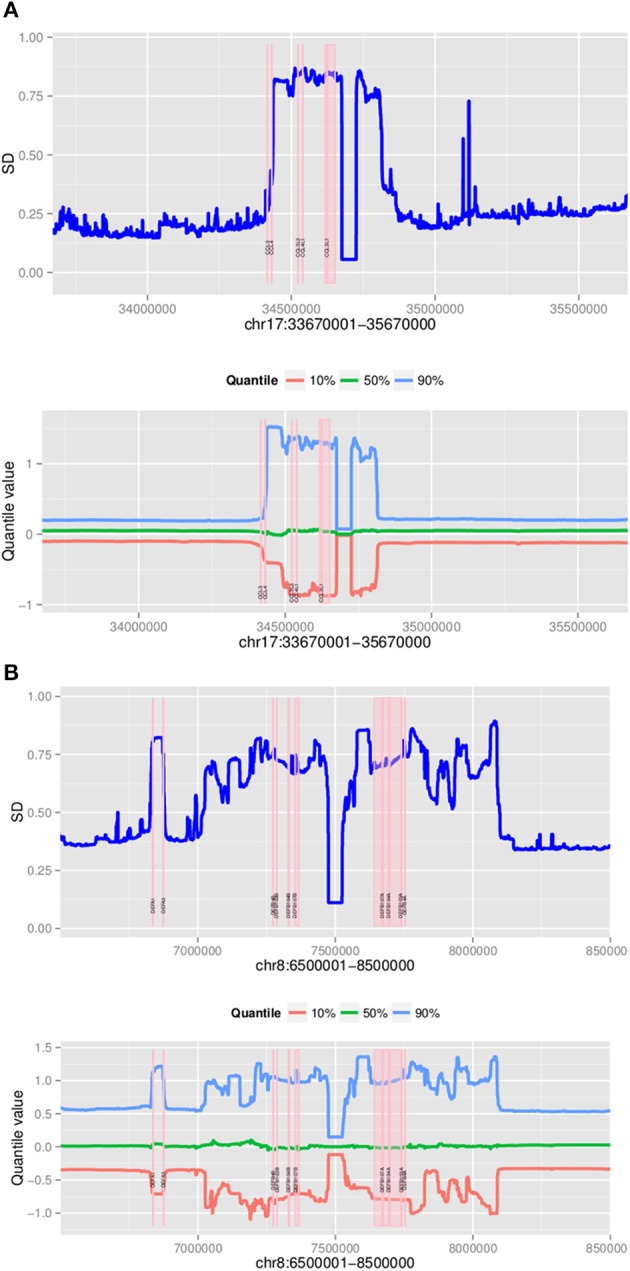
**Plots of polymorphic regions encompassing *CCL3L1* (A) and *DEFB103A* (B)**. The plots show standard deviation (top) and different percentiles (bottom) across 2 Mb sub-regions (for all 2535 samples).

**Figure 8:**
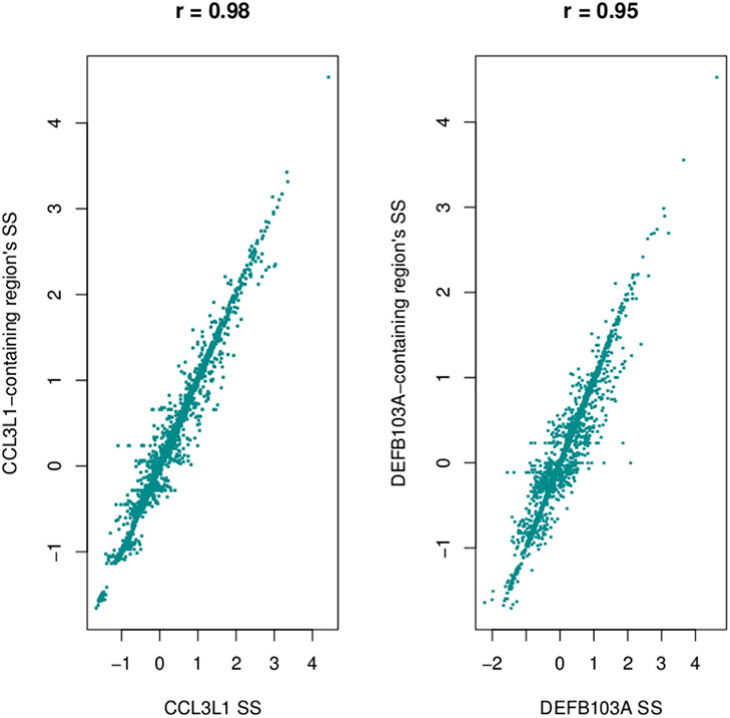
**Correlation between segmentation scores of *CCL3L1/DEFB103A* and polymorphic regions encompassing the genes**.

Using a 2 Mb region it took approximately 3 h to assign copy number at *CCL3L1* on 2535 samples using a 4-core computer with 8 Gb memory. Standard deviations and different percentiles of the segmentation results of sub-regions on the 2-Mb region were calculated to visually detect this region. Using a standard deviation threshold of 0.5, we identified *CCL3L1* within a large polymorphic CN variable region of approximately 329 kb (chr17:34436001-34815000, which included a 50 kb gap) (Figure 7). This region included small CNV blocks of clearly defined increased standard deviation (SD) which suggested that there would be multiple recombination events inside the CNV region. *CCL3* was outside this region, *CCL4* was at the boundary, while *CCL3L1* was in a second block with *CCL3L3* and *CCL4L1*. The CCL3L1 gene was located in a sub-region which had SDs fluctuating around 0.84 and 0.85. *CCL3L3* and *CCL4L1* were also in a sub-region with SDs fluctuating around 0.83 and 0.86, but these sub-regions were separated from the CCL3L1 gene-containing sub-regions by a decrease (0.8) in SDs between two blocks (Figure 7). We merged these sub-regions and the boundaries of the CCL3L1-containing region were chr17:34617501-34652500. Using CNVrd2, we obtained segmentation scores for both the CCL3L1 gene-containing region and the larger CCL3L1 region. The segmentation scores of the two regions were strongly correlated (*r* = 0.98, Figure 8).

### Assigning CCL3L1 copy number

**Figure 9:**
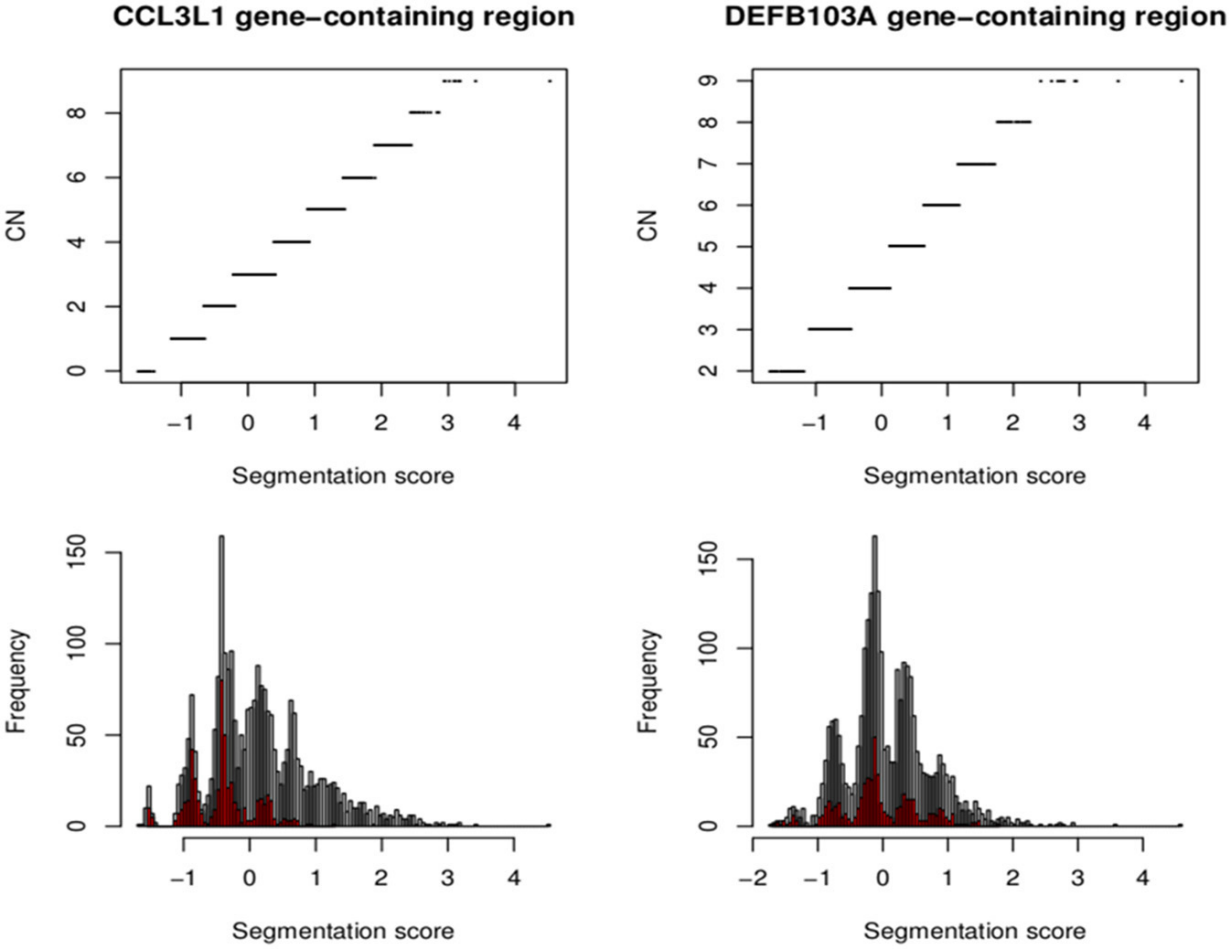
**Segmentation scores and CN groups at the CCL3L1 and DEFB103A loci**. Segmentation scores of all populations (bottom). Small populations (European for CCL3L1 and South Asian for DEFB103A) are in red. The top pictures show segmentation scores and their CN groups.

The European *CCL3L1* segmentation results from the smaller 35kb gene-containing region were assigned by CNVrd2 to five groups (Figure 9). All clusters were clearly delineated, although the highest group exhibited some scatter. The results obtained from the EM algorithm applied to the European samples were used as prior information to obtain *CCL3L1* CN estimates for the 2535 samples from the 1000 Genomes Project. The final segmentation scores across all samples ranged from −1.66 to 4.53 (Figure 9), with only one score being larger than 3.42. This value was considered to be an outlier, and the associated sample (HG00620 in the CHS population) was assigned to the largest copy number group with a probability of 1. Thus, the segmentation score range used to determine the number of groups was between −1.66 and 3.42. The distances between the groups in the European population ranged between 0.39 and 0.62. As a result, 10 groups were chosen to encompass the range of *CN* values for the full collection of samples. The Markov Chain Monte Carlo chain was convergent after 10,000 iterations and the segmentation scores were clustered into 10 groups corresponding to CN from 0 to ≥9 (Figure 9).

The CN assignments for European-ancestry populations ranged between 0 and 5, with the highest frequency (43.6%, 220/505) being 2 copies (Table 1). Similar to the European-ancestry populations, *CN* = 2 was the most common (42.1%; 208/494 samples) in the South Asian sample set. The East Asian sample set had *CN* between 0 and ≥9, with the most common being *CN* = 3 (36.7%, 189/515). *CN* = 3 was also the most frequent in the Americas sample set, with the highest *CN* = 8. The African sample sets had no individuals having 0 copies and just 10 samples (1.5%) having 1 copy, with 3–5 copies being the most common (Table 1).

### Validation of CCL3L1 CN assignments with paralog ratio test (PRT) data

The CNVrd2 results on 180 of the samples were compared to data where *CCL3L1* CN had previously been determined using PRT—45 European samples measured by Carpenter et al. (2011) and 135 African and Asian samples measured by Janyakhantikul et al. (2010). There was 77.8% (140/180) identity between the two methods (Figure 3A). The majority of discordant results were for high CN samples and differences were of one CN.

### Comparison of CNVrd2 with other read depth-based methods at CCL3L1

The packages CNVnator (Abyzov et al., 2011) and cn.Mops (Klambauer et al., 2012) were compared to CNVrd2 (refer also to Subjects and Methods). In addition, we also used the 159 genome data set of Sudmant et al. (2010). Copy number for samples in this data set were measured using a read depth-based method, but the authors used the mrsFAST aligner (Hach et al., 2010) which obtains all possible positions of a read. The concordance rates for CNVnator, cn.Mops, and the approach of Sudmant et al. (2010) with *CCL3L1* PRT-based results were 7.2% (13/180, *r* = 0.95), 36.7% (66/180, *r* = 0.65), and 0% (0/111, *r* = 0.93) respectively (Figure 3). The highest CNs called by CNVnator and cn.Mops were only 5 and 4 respectively. CN assignments of Sudmant et al. (2010) were significantly higher than the modified PRT results, although assignments of the two methods were highly correlated (*r* = 0.93) (Figure 3).

**Figure 10:**
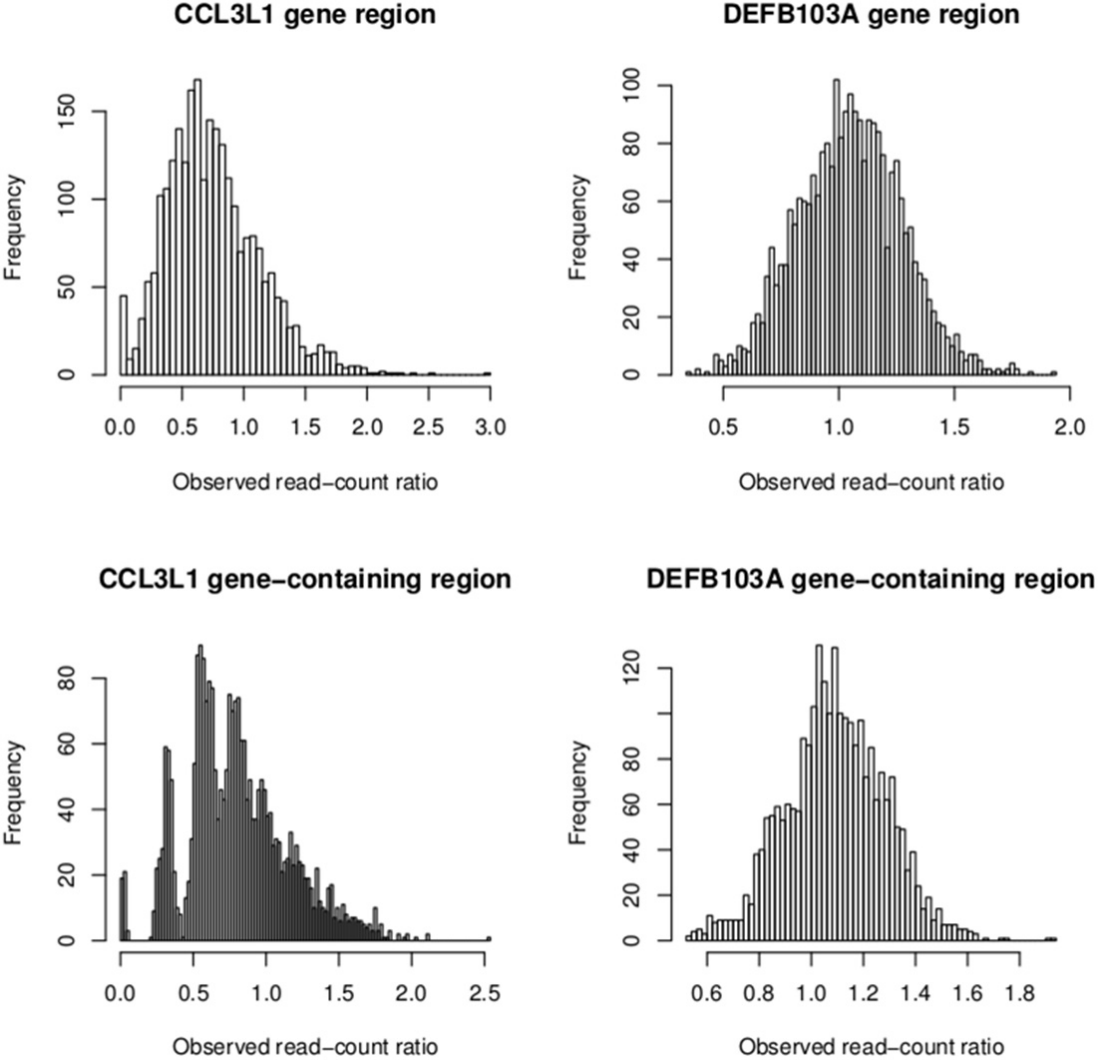
**Observed read-count ratios of samples at the *CCL3L1/DEFB103A* gene (medians: 0.7 and 1 respectively) and the *CCL3L1*/DEFB103A-containing region (medians: 0.8 and 1 respectively)**.

To investigate the discordant results of the other packages with the PRT-based method at *CCL3L1*, observed read-count ratios of European sample sets and all 2535 samples were calculated for both the CCL3L1 gene region and *CCL3L1*-containing region (Figure 10). The two medians of these ratios were considerably less than 1 (0.7 and 0.8, respectively), while the expected median was 1. For the larger *CCL3L1*-containing region, 74.7% (1893/2535) of samples had ratio <1, 76.4% (1938/2535) for the 35kb *CCL3L1* region. Reads of samples having zero copies which were aligned to CCL3L1 gene region were very close to zero (Table 4), and thus the density of reads at *CCL3L1* was not as high as expected. Approaches based solely on read depth with no cross-sample standardization would therefore have difficulty in accurately assigning *CCL3L1* CN, especially for high-CN samples, which would be assigned to lower-CN groups.

**Table 4:** **Read counts at the *CCL3L1* gene (chr17:34623842-34625730) (1.9 kb) of samples called 0 copies by CNVrd2**.

**Sample ID**	**Average coverage (2 Mb)**	**Read count at *CCL3L1***	**Read count at *CCL3L1*-containing region**	**Population**
HG00240	7.3	1	75	GBR
HG00290	7.4	0	36	FIN
HG00336	3.4	0	35	FIN
HG00410	10.1	0	69	CHS
HG00553	3.3	0	29	PUR
HG01112	4.8	0	37	CLM
HG01204	4.8	0	109	PUR
HG01260	7.2	1	40	CLM
HG01280	8.1	0	23	CLM
HG01286	4.6	4	68	PUR
HG01302	5.4	0	23	PUR
HG01474	5.1	0	40	CLM
HG01489	5.2	0	58	CLM
HG01504	5.3	0	42	IBS
HG01506	6.4	0	30	IBS
HG01550	4.7	0	38	CLM
HG01767	8.3	0	42	IBS
HG01864	8.4	0	64	KHV
HG01873	20.2	0	103	KHV
HG02122	6.2	0	58	KHV
HG02385	4.6	0	19	CDX
HG02604	7.1	2	78	PJL
HG02648	6.9	0	37	PJL
HG02652	6.9	0	45	PJL
HG02658	8.8	3	127	PJL
HG03589	5.8	0	48	BEB
HG03673	8.6	2	68	STU
HG03968	5.4	2	34	ITU
HG04019	5.6	0	39	ITU
HG04062	4.0	0	21	ITU
HG04156	5.4	0	32	BEB
NA07056	5.8	0	54	CEU
NA11831	5.8	1	67	CEU
NA18574	4.7	0	66	CHB
NA20507	6.1	0	49	TSI
NA20540	3.9	0	67	TSI
NA20589	7.0	0	41	TSI
NA20754	8.2	0	33	TSI
NA20762	7.0	0	42	TSI
NA20764	8.3	1	44	TSI
NA20778	8.3	0	78	TSI
NA20850	4.1	0	30	GIH
NA20903	7.7	0	43	GIH

*The average coverage is calculated for the 2 Mb region (chr17:33670000-35670000)*.

### Polymorphism of the DEFB103A locus

The DEFB103A region lies within a polymorphic inversion with complex structure (Sugawara et al., 2003). We used quantile values and standard deviations across sub-regions to visually identify the boundaries of the polymorphism encompassing the gene. Using a standard deviation threshold of 0.5, *DEFB103A* was identified within a large CN polymorphic region (consistent with Groth et al., 2008) of 1078 kb (chr8:70200000-7474000 7532001-8098000 including a 58 kb gap) (Figure 7). This CNV region also included small polymorphic blocks which suggested that there would be multiple recombination events inside the CNV region. *DEFB103A* was in the same block as *DEFB104A* and *DEFB107A*. The standard deviations of this block fluctuated around 0.70 and 0.74. We merged these sub-regions and the boundaries of the DEFB103A gene-containing region were chr8:7641001-7742000. The segmentation scores of this region and the larger DEFB103A gene region were strongly correlated (*r* = 0.95, Figure 8). We used this region to calculate CN for *DEFB103A*. The DEFB4A gene was outside this block owing to a relatively high peak (SD was 0.81 whilst the maximum SD of the DEFB103A gene-containing region was only 0.74) between *DEFB4A* and the *DEFB103A* block (Figure 7).

**Figure 11:**
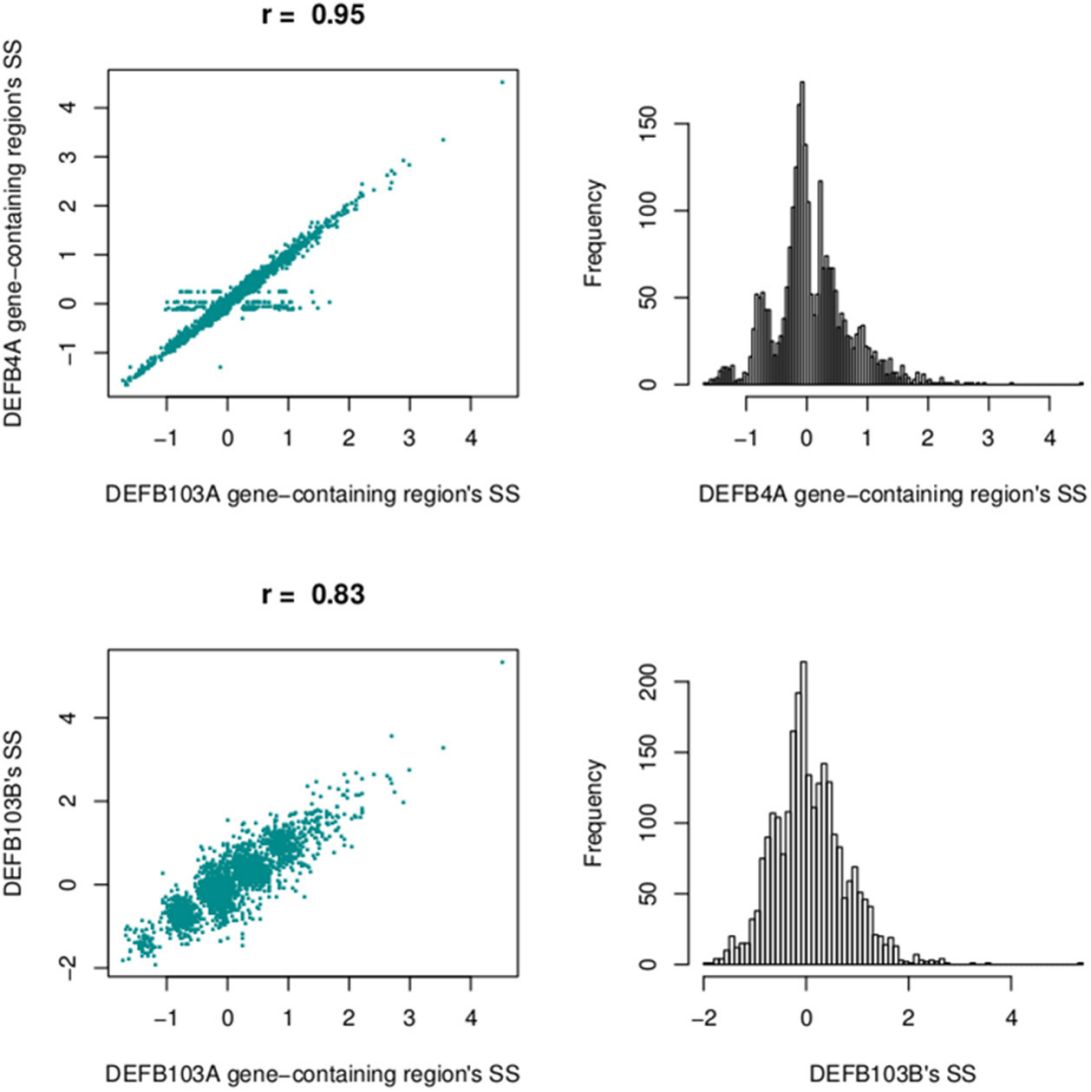
**Correlation between segmentation scores of the DEFB103A gene-containing region and the enlarged region encompassing *DEFB4A* (top left) and the DEFB103B gene region (bottom left)**. On the right are histograms of segmentation scores of the enlarged region **(top)** and DEFB103B region **(bottom)**.

We also calculated segmentation scores of *DEFB103B* (paralog of *DEFB103A*) to compare segmentation scores of this gene and those of the DEFB103A gene-containing region. The segmentation scores of the two regions were not as highly correlated (*r* = 0.83), but they made clear clusters of distinct groups (Figure 11).

### Assignment of DEFB103A CN

The segmentation scores of the gene had clear clusters (Figure 9) with some scattered values >3, therefore these values were allocated into the largest (>3) group. The South Asian population had the clearest clusters therefore we used this population to obtain prior information and ran the EM algorithm upon the segmentation score with six groups. The distances between the groups were 0.50–0.69 therefore 0.57 was set as the prior distance. Eight groups were defined for all segmentation scores and CNVrd2 was used to assign *DEFB103A* copy-number using similar parameters as for *CCL3L1* in the Bayesian clustering process (Table 2). Because the observed read count ratio range was reasonable (0.53–1.93) (Figure 11), this suggested that the lowest CN was greater than zero, with the scattering of values on the left (Figure 9) assigned to the smallest group (*CN* = 2) during the clustering process. A CN of 4 was the most common in all populations (Table 2; 26.3–47.9%), consistent with previous reports (Armour et al., 2007; Hardwick et al., 2011).

CNVrd2 obtained high concordance with PRT-based methods (90.4%, *r* = 0.95, 94/104) (Figure 3). PRT CN assignments for 104 samples were available (Hardwick et al., 2011) that also overlapped with the samples analyzed by Sudmant et al. (2010). The concordances between Sudmant et al. (2010), CNVnator (Abyzov et al., 2011) and cn.mops (Klambauer et al., 2012) (using 10 kb windows for cn.mops) with PRT-based methods were 54.8% (57/104), 1% (1/104) and 4.8% (5/104) respectively. The majority of differences between CNVrd2, Sudmant et al. (2010) and the PRT-based methods were one copy.

## Discussion

The CNVrd2 package, depicted in Figure 1, is an improved version of CNVrd previously used to assign CN at the *FCGR* locus (Nguyen et al., 2013). CNVrd2 was designed to correct issues that can occur during the segmentation process which can result in CNVrd assigning a segmentation score of zero, resulting in some samples being assigned to unreasonable CN groups. In addition, in CNVrd2, a linear regression model was used to adjust the differentiation in CN between populations. Clustering data into different groups is challenging at complex loci with high ranges of CN. Therefore, a Bayesian clustering strategy was integrated into CNVrd2 in order to obtain more reliable results at *CCL3L1* and *DEFB103A*. Finally, plots of percentiles and standard deviations of segmentation results were also added to CNVrd2. These can be used as visual tools to identify copy-number variable regions encompassing loci being measured (Figure 7). In evaluating our results, it should be noted that CNVrd2 was developed on the *FCGR* and *CCL3L1* loci. Therefore, the concordance comparisons are biased toward CNVrd2. However *DEFB103A* can be regarded as a genuine test locus, on which CNVrd2 performed well (Figure 3). There is a paucity of loci for which published PRT gold-standard data are available on complex CN loci.

CNVrd2 obtains higher concordance with PRT-based methods than other CN-assigning approaches. However the concordance was not 100% (Figure 3; 77.8% at *CCL3L1* and 90.4% at *DEFB103A*), which would limit its use in case-control analysis of complex CN loci in common disease. We used BWA alignments from the 1000 Genomes Project, where the majority of mapping qualities were zero owing to reads having multiple hits within segmental duplications. Further improvement of CNVrd2 could come from the use of alignments better optimized to detect CNV, such as mrsFAST (Hach et al., 2010) which allows diverged paralogs (1–2% difference) to be differentiated. It is important also to stress that CNVrd2 was developed to measure CN at specified loci, while other comparison packages are more automated approaches that both detect CNV and assign CN. CNVrd2 is automated to produce individual segmentation scores at a locus and assign CN using the Bayesian Information Criterion (Schwarz, 1978). However it is better to manually undertake the clustering process as we did in this study. The reason is that clear clusters were not seen at complex loci *CCL3L1* and *DEFB103A*. Note that CNVrd2 is enhanced by some prior knowledge of CN.

From the observed read count ratio analysis (Figure 10), it can be seen that if only the read-count information from one sample is used to assign *CN* (as is done by CNVnator) the majority of samples at the *CCL3L1* locus would likely be called as deletions because of the inherently low number of reads in that region, possibly resulting from *CCL3L1* reads mapping to paralogs nearby. The majority of populations had observed read-count ratios of less than 1. This could also be the reason that the cn.Mops package did not call high *CN* for multiple samples (Figure 3). Complete discordance was seen between the Sudmant et al. (2010) *CCL3L1* results obtained using multiple hits of a read and all other methods (Figure 3). The results of Sudmant et al. (2010) had been validated by PCR-based methods with good correlation. It is important to note that PCR-based methods are unreliable in measuring complex gene *CN* (Walker et al., 2009; McKinney and Merriman, 2012)—any differences in amplification efficiency that alters the ratio between the test and reference genes will alter the apparent *CN* (Armour et al., 2007). One possible reason for the Sudmant et al. (2010) discordance could be the use of different clustering strategies, but it could also be due to using different alignment methods when there are >1 read-mapping possibilities: choosing a random position (CNVrd2) vs. using all possible positions for a read (Sudmant et al., 2010). We checked the only sample (NA11831) called *CN* = 1 by Sudmant et al. (2010) and zero by CNVrd2, cn.Mops, CNVnator, and PRT-based methods. There was only one read aligned to *CCL3L1* (Table 4), while the average coverage for this sample on the 2 Mb region was adequate (5.8×). Similarly, checking all samples called as 0 copies by CNVrd2, we also saw that the number of reads aligned to the *CCL3L1* gene region were extremely low (Table 4). This suggests that the sample NA11831 was correctly assigned *CN* = 0 for *CCL3L1* by the PRT methods and the BWA aligner based next-generation sequencing methods (CNVrd2, CNVnator, cn.Mops).

At the beta-defensin locus, we identified a CNV region which had nearly equal standard deviations of sub-regions. This CNV region encompassed DEFB103A. The segmentation scores of this and the *DEFB103B* region (Figure 7; on the polymorphic section before the gap) were moderately correlated (*r* = 0.83) but they made clear clusters of different groups (Figure 11). This suggested that individual CNs of the two regions would be nearly identical. However, the identified region did not include the DEFB4A gene because we saw a high peak between the region and DEFB4A gene (Figure 7). We retested with different windows (500 and 2000 bp) and still observed this peak (data not shown). An enlarged region that included the peak and *DEFB4A* was strongly correlated with the smaller region (*r* = 0.95) but had multiple samples with a score of zero, because in CNVrd2 if a region was segmented into different sub-regions having different signs then the segmentation score of the region was assigned to zero. This suggested that there was not full duplication of the enlarged region (Figure 11). This situation could occur because of an error in the alignment process, or because there were heterogenous breakpoints within the enlarged region.

At the complex *CCL3L1* and *DEFB103A* loci CNVrd2 assigned CN with greater accuracy than the other read depth-based CN assignment methods CNVnator and cn.Mops. The CNVrd2 package is implemented in R (as of version 3.0.1) and is available from Bioconductor (as of version 2.14) at http://www.bioconductor.org/packages/release/bioc/html/CNVrd2.html, where installation and usage instructions can be found.

## Author contributions

All authors made substantial contributions to the conception or design of the work and the acquisition, analysis, or interpretation of data for the work, drafting of the work and critical revision. All authors approved the final version and agree to be accountable for all aspects of the work.

### Conflict of interest statement

The authors declare that the research was conducted in the absence of any commercial or financial relationships that could be construed as a potential conflict of interest.
